# Is heart failure with mid range ejection fraction (HFmrEF) a distinct clinical entity or an overlap group?^☆^

**DOI:** 10.1016/j.ijcha.2018.06.001

**Published:** 2018-09-06

**Authors:** Jessica Webb, Jane Draper, Lauren Fovargue, Ben Sieniewicz, Justin Gould, Simon Claridge, Carys Barton, Silapiya Smith, Kristin Tondel, Ronak Rajani, Stamatis Kapetanakis, Christopher A. Rinaldi, Theresa A. McDonagh, Reza Razavi, Gerald Carr-White

**Affiliations:** a Department of Cardiology, Guy's and St Thomas' NHS Foundation Trust, London, SE1 7EH, United Kingdom; b Division of Imaging Sciences and Biomedical Engineering, King's College London, SE1 7EH, United Kingdom; c Department of Cardiology, King's College Hospital NHS Foundation Trust, London, SE5 9RS, United Kingdom; d Division for Methods, Data Collection and Methods, Statistics Norway, Oslo, Norway; e Dep. of Mathematical Sciences and Technology, Norwegian University of Life Sciences, Ås, Norway

**Keywords:** HFrEF, Heart Failure with reduced Ejection Fraction, HFmrEF, Heart Failure with mid range Ejection Fraction, HFpEF, Heart Failure with preserved Ejection Fraction, NTproBNP, N terminal pro-B-type natriuretic peptide, Transition

## Abstract

**Background:**

The new category of heart failure (HF), Heart Failure with mid range Ejection Fraction (HFmrEF) has recently been proposed with recent publications reporting that HFmrEF represents a transitional phase. The aim of this study was to determine the prevalence and clinical characteristics of patients with HFmrEF and to establish what proportion of patients transitioned to other types of HF, and how this affected clinical outcomes.

**Methods and results:**

Patients were diagnosed with HF according to the 2016 ESC guidelines. Clinical outcomes and variables were recorded for all consecutive in-patients referred to the heart failure service. In total, 677 patients with new HF were identified; 25.6% with HFpEF, 21% with HFmrEF and 53.5% with HFrEF. While clinical characteristics and prognostic factors of HFmrEF were intermediate between HFrEF and HFpEF, HFmrEF patients had the best outcome, with higher mortality in the HFrEF population (p 0.02) and higher HF rehospitalisation rates in the HFpEF population (*p* < 0.01).

38.7% of the HFmrEF patients transitioned (56.4% to HFpEF and 43.6% to HFrEF) with fewest deaths in the patients that transitioned to HFpEF (p 0.04), and fewest HF readmissions in the patients that remained as HFmrEF (<0.01)

**Conclusion:**

HFmrEF patients had the best outcomes, compared to high rates of mortality seen in patients with HFrEF and high rates of HF readmissions seen in patients with HFpEF. Only 1/3 of HFmrEF patients transitioned during follow up, with the lowest mortality seen in patients transitioning to HFpEF.

## 1 Introduction

Effective therapies to date have only been demonstrated in heart failure (HF) patients with a left ventricular ejection fraction (LVEF) ≤ 35–40% [[1], [2], [3]] and subsequently, current HF guidelines have set this at the cut off for Heart Failure with reduced ejection Fraction (HFrEF) [4, 5]. In 2016, the European Society of Cardiology introduced the category, Heart Failure with mid range Ejection Fraction (HFmrEF) in order to acknowledge the ‘grey area’ between HFrEF and Heart Failure with preserved Ejection Fraction (HFpEF) and to improve identification of the latter, as these patients are more challenging to diagnose [5, 6]. Whereas it is well accepted that HFrEF and HFpEF differ with respect to underlying aetiologies, demographics and comorbidities [7], there has been uncertainty with respect to the HFmrEF patients. It has been reported that these patients are similar to HFrEF patients [8, 9] and also that HFmrEF patients are more similar to HFpEF with no differences in mortality or HF hospitalisation [10]. Moreover, it has been published that HFmrEF patients represent an overlap phase with a high rate of patients transitioning to HFrEF and HFpEF [11] with improved outcomes when transitioning to HFpEF [12]. The exact prevalence of HFpEF in the United Kingdom remains uncertain [[13], [14], [15]] and it is not clear what number of patients experience HFmrEF, which then poses challenges for trials, clinical management and workforce planning.

This study sought to determine the prevalence and clinical characteristics of patients with HFmrEF in a large unselected heart failure population and to establish what proportion of patients transitioned to other types of HF, and how this affected clinical outcomes.

## 2 Methods

All consecutive patients who had acute presentations of suspected decompensated HF and raised plasma NT-proBNP tested in our institution over one year were included, between 10/09/2014 and 09/09/2015. Patients were diagnosed with HF during their first HF admission, after an expert physician review according to the 2016 ESC guidelines [4], with signs and symptoms of HF, raised NTproBNP measured at index presentation and echocardiography to establish left ventricular ejection fraction (LVEF) and evidence of structural heart disease or diastolic dysfunction. Patients were subsequently categorised as HFrEF, HFpEF or HFmrEF if their LVEF was <40%, >50% or 40–49%, respectively. In case of uncertainty, diagnoses were adjudicated through the heart failure multidisciplinary team. Follow up echocardiography was performed at the clinician's discretion This was a retrospective study.

Hospital databases and medical records were used to confirm symptoms, patient demographics, risk factors, length of stay, time to heart failure hospitalisation and mortality. Outpatient mortality was confirmed using Summary Care Records. When patients were admitted more than once, their first chronological presentation was recorded during the study period. All patients with HFmrEF had all their previous and subsequent echocardiogram examinations reviewed to establish if these patients had transitioned. The time to the second echo was recorded. HFmrEF patients who did not have a follow uo echo performed were excluded from the transition analysis. Left atrial enlargement (LAE) was defined on the parasternal long axis echocardiographic images as over 38 mm in women and 40 mm in men or left atrial volume of over 52 mls/m^2^ and 58 mls/m^2^, respectively. Left ventricular hypertrophy (LVH) was defined as myocardial wall thickness on echocardiography of >12 mm in the parasternal long axis views. Data was collected as part of our Institution's approved Clinical Audit.

Continuous variables are described with mean ± standard deviation (SD) for normally distributed variables and median and interquartile range for non-normally distributed variables. Categorical variables are described as frequencies and percentages. Associations between baseline variables were evaluated using analysis of variance, Mann-Whitney U *t*-test and chi-square tests, where appropriate. Survival data were assessed using Kaplan Meier analysis. Multivariable cox analysis was performed using different variables (age, NTproBNP, LVEF and number of risk factors) to establish if there were differences between the different categories of HF. Statistical significance was defined as a *p* value of <0.05.

## 3 Results

### 3.1 Baseline characteristics

**Fig. 1:**
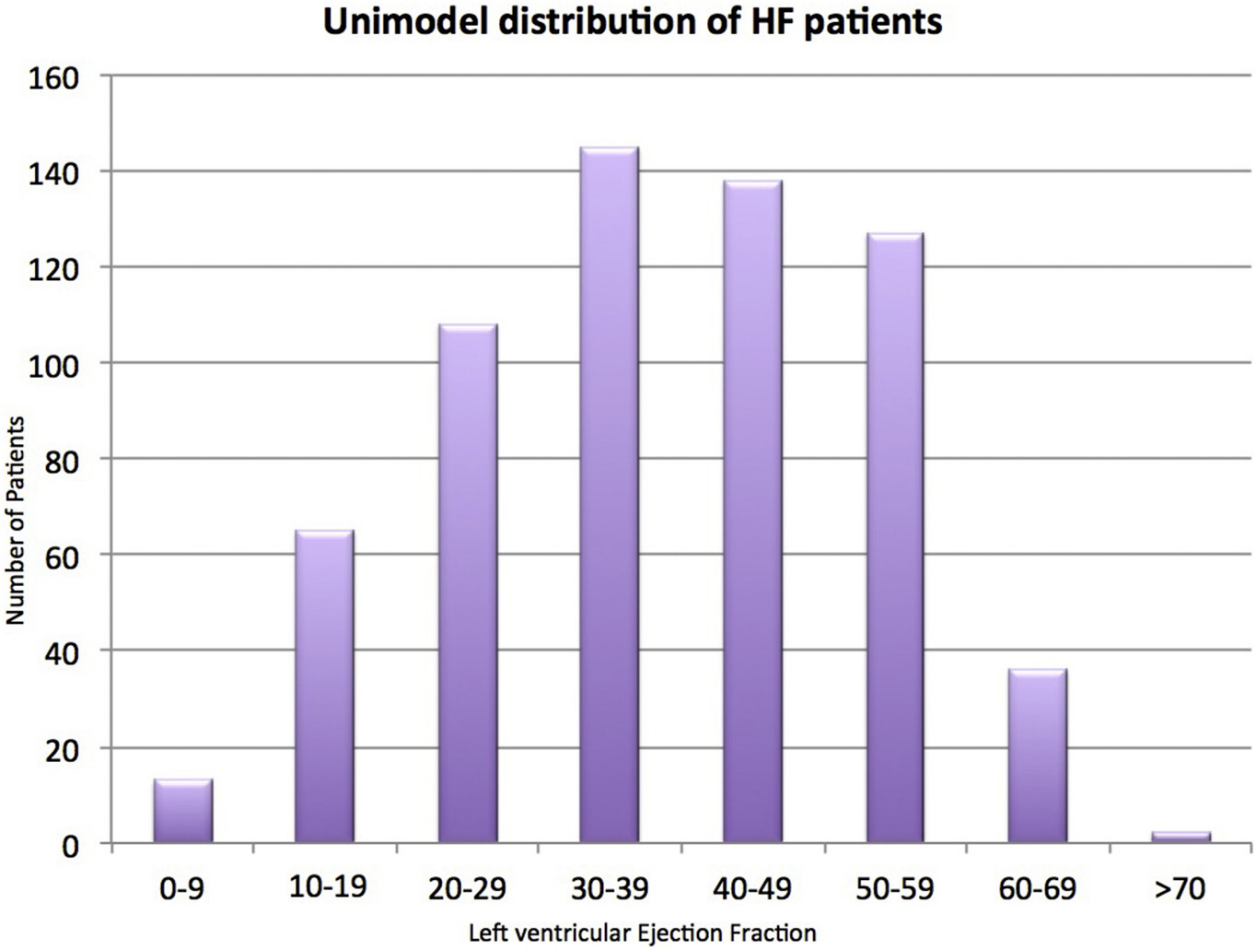
Distribution of LVEF for all heart failure patients admitted to our institution This bar chart shows a unimodel distribution of LVEF for all patients admitted with heart failure.

Overall, 677 patients with new HF were identified; 173 patients with HFpEF (25.6%), 142 patients with HFmrEF (21%) and 362 patients with HFrEF (53.5%). The distribution of LVEF for all HF patients is shown in Fig. 1.

Patients with HFrEF were younger, more likely to be male and caucasian with a higher NTproBNP (Table 1). Patients with HFpEF were more older, more likely to be female and of AfroCaribbean origin, with the lowest NTproBNP and glomerular filtration rate (GFR). These patients had the greatest number of risk factors. Patients with HFmrEF were statistically different to those with HFpEF with respect to age, gender, ethnicity, blood results (NTproBNP, PCV, albumin) and risk factor profile. When compared to the HFrEF population, patients with HFmrEF were statistically different with respect to age, gender, length of stay, bloods (haemoglobin, PCV, NTproBNP) and presence of hypertension and obstructive sleep apnoea. On multivariate analysis, NTproBNP, age, LVEF and number of risk factors were significant for the category of heart failure (*p* < 0.05).

**Table 1:** Clinical characteristics of all HF patients.

	All HF patients	HFrEF (A)	HFmrEF (B)	HFpEF (C)	p A v B	p A v C	p B v C
Age (years)	72.8 ± 14.2	70.0 ± 15.1	74.5 ± 13.3	76.7 ± 11.8	*p* < 0.05	p < 0.05	NS
Male (%)	58.6	69.7	52.2	42.3	p < 0.05	p < 0.05	NS
LVEF	39.2 (22.5)	27.2 (11.8)	44.0 (5)	56.2 (2.5)	p < 0.05	p < 0.05	p < 0.05
Caucasian (%)	68.2	72.1	64.7	63.5	NS	NS	NS
AfroCaribbean (%)	16.5	11.7	16.9	25.3	NS	p < 0.05	p < 0.05
Asian (%)	6.6	5.2	9.6	7.1	NS	NS	NS
Length of stay (days)	9(14)	10 (14.5)	8 (12.25)	7 (14)	p < 0.05	p < 0.05	NS
Haemoglobin (g/l)	115.8 ± 20.4	117.1 ± 21.5	109.4 ± 14.7	115.2 ± 19.5	p < 0.05	p < 0.05	NS
MCV	91.7 ± 7.1	92 ± 6.8	90.8 ± 9.4	90.9 ± 6.5	NS	NS	NS
PCV	0.358 ± 0.06	0.362 ± 0.06	0.339 ± 0.04	0.355 ± 0.06	p < 0.05	NS	p < 0.05
Plasma Sodium	137.9 ± 4.7	137.8 ± 4.8	138.3 ± 3.6	138.0 ± 5.1	NS	NS	NS
GFR (ml/min/1.73cm^2^)	59.1 ± 30.5	61.5 ± 30.9	55.8 ± 28.9	49.4 ± 30.8	NS	p < 0.05	NS
Albumin	36.8 ± 6.7	37.4 ± 6.5	36.5 ± 7.8	34.1 ± 5.2	NS	p < 0.05	p < 0.05
NTproBNP (pg/ml)	4273 (9201)	6416 (13198)	4246 (7894)	2344 (4753)	p < 0.05	p < 0.05	p < 0.05
Mean number of comorbidities	3.0 ± 1.7	2.7 ± 1.6	3.0 ± 1.6	3.7 ± 1.6	NS	*p* < 0.05	p < 0.05
Atrial Fibrillation or Flutter (%)*	43.0	42.0	37.5	49.4	NS	NS	p < 0.05
Diabetes (%)	43.7	39.0	44.9	51.8	NS	p < 0.05	NS
Hypertension (%)	64.7	54.6	67.6	81.8	p < 0.05	p < 0.05	p < 0.05
COPD (%)	31.8	29.1	30.9	37.6	NS	NS	NS
Coronary Artery Disease (%)	42.7	45.7	44.1	35.9	NS	p < 0.05	NS
Hypercholesterolaemia (%)	40.7	35.3	37.5	53.5	NS	p < 0.05	p < 0.05
Obesity (BMI ≥ 30 kg/m^2^) (%)	20.9	12.3	18.4	39.4	NS	p < 0.05	p < 0.05
Obstructive Sleep Apnoea (%)	5.9	2.8	8.8	9.4	p < 0.05	p < 0.05	NS
History of Cerebrovascular accident (%)	10.9	10.4	10.3	12.4	NS	NS	NS
Iron deficiency anaemia (%)	31.2	28.8	25.0	40.6	NS	p < 0.05	p < 0.05

NS Not significant; LVEF: left ventricular ejection Fraction; GFR: Glomerular filtration rate (mls/min); AF: atrial fibrillation; IHD: Ischaemic heart Disease; COPD Chronic Obstructive pulmonary disease; ACE/ARB: Angiotensin converting enzyme inhibitor/angiotensin II receptor blockers.

### 3.2 Association between heart failure category and outcome

During follow up, median 26.8 months (range 22.1–34.0) there were 270 patients who died. Follow up was 100% complete. 40% of the HFrEF patients died compared to 28% of the HFmrEF patients and 34% of the HFpEF patients (p 0.02, p 0.01, p 0.07 respectively).

**Fig. 2:**
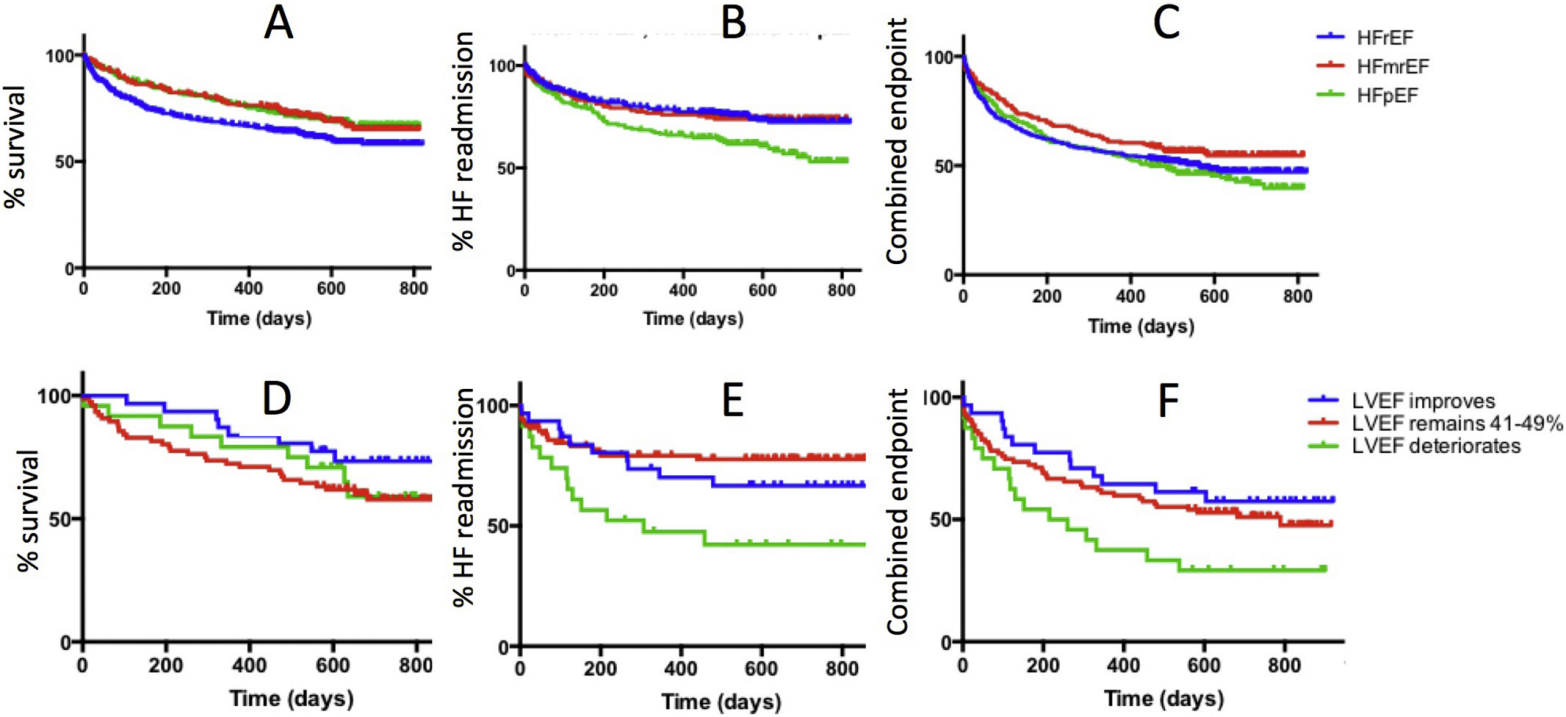
A: Kaplan Meier survival free of all cause death for all HF patients; *p* = 0.0256 HFrEF v HFpEF; 0.0298 HFrEF v HFmrEF, NS HFpEF v HFmrEF B: Kaplan Meier for time to HF readmissions for all HF patients; *p* = 0.0012 HFpEF v HFrEF, NS HFmrEF v HFrEF, *p* = 0.009 HFmrEF v HFpEF C: Kaplan Meier for time to combined endpoint (all cause death/HF readmissions) for all HF patients; *p* = 0.0399 HFpEF v HFmrEF, NS HFrEF v HFmrEF, NS HFrEF v HFpEF D: Kaplan Meier for time to all cause death in all patients with HFmrEF; *p* = 0.04 LVEF remain v improve, *p* = 0.28 remain v deteriorate, *p* = 0.56 improve v deteriorate E: Kaplan Meier for time to HF readmissions in all patients with HFmrEF; *p* = 0.56 LVEF remain v improve, *p* < 0.01 remain v deteriorate, *p* = 0.04 improve v deteriorate F: Kaplan Meier for time to combined endpoint (all cause death/HF readmissions) in all patients with HFmrEF; *p* = 0.36 LVEF remain v improve, p 0.06 remain v deteriorate, *p* = 0.02 improve v deteriorate.

The Kaplan-Meier survival curves for all cause death, time to HF readmission and composite end point (HF readmission and all cause death) are shown in Fig. 2A, B and C. Statistical differences were shown for all cause mortality (HFrEF v HFmrEF, HFrEF v HFpEF, p 0.02), HF readmission (HFrEF v HFmrEF, HFmrEF v HFpEF, *p* < 0.01).

### 3.3 Proportion of HFmrEF patients who transitioned during follow up

In total 114 out of the 142 (80.3%) HFmrEF patients had follow up echocardiography performed (median 13.1, IQR 4.2–32.4 months). 38.7% of the HFmrEF patients transitioned during follow up (56.4% to HFpEF and 43.6% to HFrEF, Fig. 2D, E and F) with the clinical demographics in Table 2. 8 out of the 14 patients that improved their LVEF during follow up underwent successful revascularisation. It was difficult to clinically differentiate the patients, although patients who transitioned to HFrEF were more likely to have LAE and had a tendency to have atrial fibrillation and more comorbidities. Note is made that patients who recovered were less likely to be discharged from hospital on betablocker therapy but more likely to be prescribed aldosterone antagonists.

**Table 2:** Clinical characteristics of HFmrEF patients who had follow up echocardiography.

	Average (*n* = 114)	HFmrEF with LVEF improving (A: *n* = 31)	HFmrEF LVEF remains 40–49% (B: *n* = 59)	HFmrEF with LVEF deteriorating (C: *n* = 24)	P A v B	P A v C	P B v C
Age	74.8 ± 13.1	75.3 ± 9.0	75.2 ± 10.2	72.6 ± 13.3	NS	NS	NS
Gender (male)	57 (50)	13 (41.9)	35 (59.3)	9 (37.5)	NS	NS	p0.03
Caucasian	74 (64.9)	21 (67.7)	39 (66.1)	14 (58.3)	NS	NS	NS
AfroCaribbean	21 (18.4)	5 (16.1)	9 (15.2)	7 (29.2)	NS	NS	NS
Asian	11 (9.6)	3 (9.7)	5 (8.5)	3 (12.5)	NS	NS	NS
LVEF	42.9 ± 2.5	43.1 ± 2.2	43.0 ± 2.6	42.2 ± 2.4	NS	NS	NS
Average E/E'	14.0 ± 7.7	15.8 ± 9.9	13.0 ± 7.2	15.1 ± 6.5	NS	NS	NS
Left atrial enlargement	57 (50)	12 (38.7)	27 (45.8)	18 (75.0)	NS	p < 0.01	p0.01
Left ventricular hypertrophy	57 (50)	13 (41.9)	27 (45.8)	12 (50.0)	NS	NS	NS
NTproBNP	4100 (7424)	4600 (6734)	4100 (7424)	4745 (6921)	NS	NS	NS
GFR	51.3 ± 26.0	45.3 ± 17.5	54.2 ± 28.8	44.8 ± 22.1	NS	NS	NS
Average number of comorbidities	3.0 ± 1.6	2.6 ± 1.7	2.9 ± 1.6	3.6 ± 1.4	NS	p0.03	NS
AF	46 (40.4)	10 (32.3)	22 (35.6)	14 (58.3)	NS	NS	p0.04
IHD	50 (43.9)	14 (45.2)	26 (44.1)	10 (41.7)	NS	NS	NS
Diabetes	50 (43.9)	13 (41.9)	24 (40.7)	13 (54.2)	NS	NS	NS
Hypertension	71 (62.3)	16 (51.6)	38 (64.4)	19 (79.2)	NS	p0.03	NS
COPD	34 (29.8)	7 (22.6)	17 (32.2)	10 (41.7)	NS	NS	NS
Hypercholesterolaemia	42 (36.8)	7 (22.6)	24 (40.7)	11 (45.8)	NS	NS	NS
Obstructive Sleep Apnoea	10 (8.58	4 (12.9)	5 (8.5)	1 (4.2)	NS	NS	NS
Obesity (BMI ≥ 30 kg/m^2^) (%)	19 (16.7)	5 (16.1)	9 (15.3)	7 (29.2)	NS	NS	NS

Medications at discharge							
ACE/ARB	67 (58.8)	16 (51.6)	36 (61.0)	13 (54.2)	NS	NS	NS
Beta blockers	62 (54.4)	5 (16.1)	42 (71.2)	15 (62.5)	p < 0.01	p < 0.01	NS
Aldosterone antagonist	39 (34.2)	20 (64.5)	11 (18.6)	8 (33.3)	p < 0.01	p0.02	NS
Diuretics	99 (86.8)	27 (87.1)	48 (81.3)	24 (100)	NS	NS	p0.03

LVEF: left ventricular ejection Fraction; GFR: Glomerular filtration rate (mls/min); AF: atrial fibrillation; IHD: Ischaemic heart Disease; COPD Chronic Obstructive pulmonary disease; ACE/ARB: Angiotensin converting enzyme inhibitor/angiotensin II receptor blockers.

Kaplan Meier graphs confirmed fewest deaths in the patients that transitioned to HFpEF (p 0.04), and fewest HF readmissions in the patients that remained as HFmrEF (<0.01).

## 4 Discussion

While clinical characteristics and prognostic factors of HFmrEF were intermediate between HFrEF and HFpEF, HFmrEF patients had the best outcome, with higher mortality in the HFrEF population and higher HF readmission rates in the HFpEF population. 38.7% of the HFmrEF patients transitioned (56.4% to HFpEF and 43.6% to HFrEF) with fewest deaths in the patients that transitioned to HFpEF, and fewest HF readmission in the patients that remained as HFmrEF.

Moreover, this study clearly defined the prevalence of both HFpEF and HFmrEF in this population, a tertiary hospital in central London. The number of patients with HFpEF was greater than previously published in the United Kingdom [15] and more than the recent National Heart Failure Audit (NICOR data) [13, 14]. This difference may be in part because of differences in methodology. Data from Southend University Hospital NHS Foundation collected in 2012 and published in 2016 reported 17% of patients admitted with heart failure had HFpEF [15]. However, of the 668 patients episodes over a year, 97 were readmissions, resulting in 571 patients of which 113 did not have echocardiography. 83 patients were excluded due to valvular disease resulting in 200 patients with HFrEF, 41 patients with HFpEF (defined as LVEF > 50% with E/e' ≥ 15) and 134 patients with heart failure symptoms, but 81 patients with LVEF > 50% and E/e' not measured and 53 patients with LVEF > 50% and E/e' ≤ 15. It is likely that the number of patients with HFpEF would have increased had all the patients had echocardiography and other echocardiographical measures were used (structural heart changes such as LVH or LAE).

The 2015/2016 NICOR data reported that out of 66,695 patients admitted with heart failure, 11.1% had diastolic dysfunction and 7.1% of the patients were reported on echo as having LVH, in contrast to 68.3% who had left ventricular systolic dysfunction [14]. Using the ESC updated HF diagnostic guidelines, this translates to 17.2% of the patients diagnosed as HFpEF although it is possible that patients with LAE may have been counted as having a normal echo (2.7%). However, this percentage is likely to be an underestimation as the audit is biased towards reporting HFrEF patients, as one of the key aims is to establish whether HFrEF patients have optimal medical therapy, and so it is possible not all patients with HFpEF were included. One of the reasons for this is because not all HFpEF patients are linked into the HF services during their admission due to practical difficulties in identifying them as they have multiple comorbidities.

The number of patients with HFmrEF was more than a recent publication in Spain that recorded 14% of a population of 3580 HF patients [12]. It is not clear why more patients at our institution were diagnosed with HFmrEF, but it is well known that geographical variations exist in the prevalence of HFpEF [[16], [17], [18]] and this may contribute to the difference reported in HFmrEF. Our data further differs from published literature as our patients with HFmrEF had fewer HF readmissions and reduced mortality that is in contrast to previous reports [9, 10, 19]. These differences are important as the HFmrEF patients are at lowest risk.

Moreover, fewer patients with HFmrEF transitioned to other types of HF than has previously been reported [11] although it was not possible to identify these patients clinically using the variables in Table 2, apart from LAE. One of the strengths of this study was that 80% of all the patients had echocardiography during follow up. The differences that we have shown to published literature in addition to geographical variation, might be in part due to the small number of patients, that more clinical parameters are needed to separate out these groups and subtle differences in disease pathology. Even though similar numbers of the HFmrEF patients had IHD, 8 out of the 14 patients who imporved LVEF during follow up underwent successful coronary revascularisation. Furthermore, it may be that these patients would benefit from phenomapping, similar to what has been described in patients with HFpEF [20]. Interestingly, our work is in agreement with the spanish publication that also shows the lowest mortality in patients that transition from HFmrEF to HFpEF.

Recent work has shown an association between HF medications and improvement in outcomes in patients with LVEF > 40% [21]. It is surprising that the HFmrEF patients who recovered systolic function during follow up were the least likely to be prescribed beta blockers and most likely to be on aldosterone. It would be interesting to test outcome and improvement in LV function in a prospective manner in a larger number of patients. Of note, all the patients that demonstrated deterioration in LVEF were discharged on diuretics.

In addition, important differences with previously published literature in our risk factor profiles do however exist although broadly our HFmrEF characteristics are similar to previously published studies, as intermediate between HFrEF and HFpEF [9, 10, 19, [22], [23], [24]]. The Adhere registry reported different risk factor profiles with HF/normal LVEF (>55%) less likely to have hyperlipidaemia and higher proportions of african americans presenting in the reduced LVEF categories [18, 25]. It has also been reported that patients with LVEF 40–55% have previous myocardial infarction and diabetes more than heart failure patients with LVEF > 55% [19, 23], and the Cardiovascular Health Study (CHS) reported higher levels of diabetes in the HFmrEF population [24].

The CHARM program [22] reported a unimodel distribution across the LVEF deciles suggesting a significant proportion of patients in the ‘middle band’ of LVEF, which is similar to what we reported in this study (Fig. 1), suggesting that even though HFmrEF is a new category in HF classification, it is not a new phenomenon. Previous reports have suggested that the estimated prevalence of this middle range group is 10–20%, which may be low partly because most patients with a mild reduction in LVEF do not have clinical heart failure. Certainly this is reflected in our data, with a similar proporion of patients with HFmrEF.

Moreover, it is widely accepted that LVEF may not be the most sensitive parameter of function [26], and measures of myocardial deformation may be more accurate [27]. Moreover, despite echocardiography being the most accessible imaging modality, there are issues with the inherent variability in the measurement of LVEF using echocardiography and that Cardiac Magnetic Resonance imaging is the gold standard at assessing volumes and function [28]. Despite these issues, LVEF has remained the main tool for classification as historically clinical studies have shown clear outcome benefits in patients with reduced LVEF [[1], [2], [3], [29], [30], [31], [32], [33]] and there is no other obviously available alternative.

What remains certain however, is that since the advent of the 2016 ESC guidelines, there have been numerous publications describing different HFmrEF patients and that these differences either reflect geographical variations or that within the HFmrEF population there remain distinct clinical clusters that need further differentiation. More work is needed to understand and validate these differences, before large scale trials can be designed to test which medications impact outcomes.

## 5 Limitations

Limitations are that NTproBNP was tested on admission and not on discharge, which would have been useful as part of inpatient risk scores and that the data was collected retrospectively. The number of patients is another potential limitation, although as important differences are seen, thes results are still important. In addition, follow up echocardiographic was performed when indicated clinically and not done at prespecified intervals which makes it difficult to compare change in function with time. There was a small number of patients who did not have echocardiography performed duing follow up. This will have contributed to a source of bias. More work is needed to establish the difference between LVEF in different imaging modalities in a prospective manner in these patients with respect to time.

## 6 Conclusions

We have characterised the HFmrEF population in a large unselected group of inner London heart failure patients, demonstrating that they are part of a unimodal LVEF distribution and a distinct clinical group with a different risk profile and better outcomes. Only 1/3 of HFmrEF patients transitioned during follow up, with the lowest mortality seen in patients transitioning to HFpEF. These findings should help in designing future studies looking at treatment options in this group.

## Abbreviations

HF: Heart failure
LVEF: Left ventricular ejection fraction
HFrEF: Heart Failure with reduced Ejection Fraction
HFmrEF: Heart Failure with mid range Ejection Fraction
HFpEF: Heart Failure with preserved Ejection Fraction
NTproBNP: N terminal pro-B-type natriuretic peptide
NYHA: New York Heart Association
ESC: European Society of Cardiology
MCV: mean corpuscular volume
PCV: packed cell volume
GFR: glomerular filtration rate
LAE: Left atrial enlargement
LVH: Left ventricular hypertrophy

## Funding

This work was supported by the Wellcome EPSRC Centre for Medical Engineering at King's College London (WT 203148/Z/16/Z).

## Conflicts of interest

The authors have no conflicts of interest to declare.

## References

[1] Pitt B., Zannad F., Remme W.J., Cody R., Castaigne A., Perez A. (1999). The effect of spironolactone on morbidity and mortality in patients with severe heart failure. Randomized Aldactone evaluation study investigators. N. Engl. J. Med..

[2] Group CTS (1987). Effects of enalapril on mortality in severe congestive heart failure. Results of the Cooperative North Scandinavian Enalapril Survival Study (CONSENSUS). N. Engl. J. Med..

[3] Packer M., Bristow M.R., Cohn J.N., Colucci W.S., Fowler M.B., Gilbert E.M. (1996). The effect of carvedilol on morbidity and mortality in patients with chronic heart failure. U.S. carvedilol heart failure study group. N. Engl. J. Med..

[4] Ponikowski P., Voors A.A., Anker S.D., Bueno H., Cleland J.G., Coats A.J. (2016). 2016 ESC guidelines for the diagnosis and treatment of acute and chronic heart failure: the task force for the diagnosis and treatment of acute and chronic heart failure of the European Society of Cardiology (ESC) developed with the special contribution of the heart failure association (HFA) of the ESC. Eur. Heart J..

[5] Yancy C.W., Jessup M., Bozkurt B., Butler J., Casey D.E., Drazner M.H. (2013). 2013 ACCF/AHA guideline for the Management of Heart Failure A report of the American College of Cardiology Foundation/American Heart Association task force on practice guidelines. J. Am. Coll. Cardiol..

[6] Mcmurray J.J., Adamopoulos S., Anker S.D., Auricchio A., Bohm M., Dickstein K. (2012). ESC guidelines for the diagnosis and treatment of acute and chronic heart failure 2012: the task force for the diagnosis and treatment of acute and chronic heart failure 2012 of the European Society of Cardiology. Developed in collaboration with the heart failure association (HFA) of the ESC. Eur. J. Heart Fail..

[7] Butler J., Fonarow G.C., Zile M.R., Lam C.S., Roessig L., Schelbert E.B. (2014). Developing therapies for heart failure with preserved ejection fraction: current state and future directions. JACC Heart Fail..

[8] Chioncel O., Lainscak M., Seferovic P.M., Anker S.D., Crespo-Leiro M.G., Harjola V.P. (2017). Epidemiology and one-year outcomes in patients with chronic heart failure and preserved, mid-range and reduced ejection fraction: an analysis of the ESC heart failure long-term registry. Eur. J. Heart Fail..

[9] Pascual-Figal D.A., Ferrero-Gregori A., Gomez-Otero I., Vazquez R., Delgado-Jimenez J., Alvarez-Garcia J. (2017). Mid-range left ventricular ejection fraction: clinical profile and cause of death in ambulatory patients with chronic heart failure. Int. J. Cardiol..

[10] Gomez-Otero I., Ferrero-Gregori A., Varela Roman A., Seijas Amigo J., Pascual-Figal D.A., Delgado Jimenez J. (2017). Mid-range ejection fraction does not permit risk stratification among patients hospitalized for heart failure. Rev Esp Cardiol (Engl Ed).

[11] Tsuji K., Sakata Y., Nochioka K., Miura M., Yamauchi T., Onose T. (2017). Characterization of heart failure patients with mid-range left ventricular ejection fraction-a report from the CHART-2 study. Eur. J. Heart Fail..

[12] Farre N., Lupon J., Roig E., Gonzalez-Costello J., Vila J., Perez S. (2017). Clinical characteristics, one-year change in ejection fraction and long-term outcomes in patients with heart failure with mid-range ejection fraction: a multicentre prospective observational study in Catalonia (Spain). BMJ Open.

[13] Akosua Donkor JC, Theresa McDonagh, Suzanna Hardman. National Heart Failure Audit NICOR (National Institute for Cardiovascular Outcomes Research). 2014/2015.

[14] Akosua Donkor TM, Suzanna Hardman. National Heart Failure Audit. NICOR (National Institute for Cardiovascular Outcomes Research). 2015/2016.

[15] Rajdip Dulai A.S.S., Qureshi Amer, Katechia Shanit, Peysakhova Yulia, Johns Moira, Mazhar Sajjad (2016). Prevalence, clinical characteristics and outcomes of HF with preserved versus reduced ejection fraction. Br. J. Cardiol..

[16] Atherton J.J., Hayward C.S., Wan Ahmad W.A., Kwok B., Jorge J., Hernandez A.F. (2012). Patient characteristics from a regional multicenter database of acute decompensated heart failure in Asia Pacific (ADHERE International-Asia Pacific). J. Card. Fail..

[17] West R., Liang L., Fonarow G.C., Kociol R., Mills R.M., O'Connor C.M. (2011). Characterization of heart failure patients with preserved ejection fraction: a comparison between ADHERE-US registry and ADHERE-International registry. Eur. J. Heart Fail..

[18] Yancy C.W., Lopatin M., Stevenson L.W., De Marco T., Fonarow G.C., Committee A.S.A. (2006). Clinical presentation, management, and in-hospital outcomes of patients admitted with acute decompensated heart failure with preserved systolic function: a report from the Acute Decompensated Heart Failure National Registry (ADHERE) database. J. Am. Coll. Cardiol..

[19] Chioncel O., Lainscak M., Seferovic P.M., Anker S.D., Crespo-Leiro M.G., Harjola V.P. (2017). Epidemiology and one-year outcomes in patients with chronic heart failure and preserved, mid-range and reduced ejection fraction: an analysis of the ESC heart failure long-term registry. Eur. J. Heart Fail..

[20] Shah S.J., Katz D.H., Selvaraj S., Burke M.A., Yancy C.W., Gheorghiade M. (2015). Phenomapping for novel classification of heart failure with preserved ejection fraction. Circulation.

[21] Zheng S.L., Chan F.T., Nabeebaccus A.A., Shah A.M., McDonagh T., Okonko D.O. (2018). Drug treatment effects on outcomes in heart failure with preserved ejection fraction: a systematic review and meta-analysis. Heart.

[22] Solomon S.D., Anavekar N., Skali H., M8 J.J., Swedberg K., Yusuf S. (2005). Influence of ejection fraction on cardiovascular outcomes in a broad spectrum of heart failure patients. Circulation.

[23] He K.L., Burkhoff D., Leng W.X., Liang Z.R., Fan L., Wang J. (2009). Comparison of ventricular structure and function in Chinese patients with heart failure and ejection fractions > 55% versus 40% to 55% versus <40%. Am. J. Cardiol..

[24] Gottdiener J.S., Mcclelland R.L., Marshall R., Shemanski L., Furberg C.D., Kitzman D.W. (2002). Outcome of congestive heart failure in elderly persons: influence of left ventricular systolic function. The cardiovascular health study. Ann. Intern. Med..

[25] Sweitzer N.K., Lopatin M., Yancy C.W., Mills R.M., Stevenson L.W. (2008). Comparison of clinical features and outcomes of patients hospitalized with heart failure and normal ejection fraction (> or =55%) versus those with mildly reduced (40% to 55%) and moderately to severely reduced (<40%) fractions. Am. J. Cardiol..

[26] Sanderson J.E. (2014). HFNEF, HFpEF, HF-PEF, or DHF: what is in an acronym?. JACC Heart Fail..

[27] Kraigher-Krainer E., Shah A.M., Gupta D.K., Santos A., Claggett B., Pieske B. (2014). Impaired systolic function by strain imaging in heart failure with preserved ejection fraction. J. Am. Coll. Cardiol..

[28] Bellenger N.G., Burgess M.I., Ray S.G., Lahiri A., Coats A.J., Cleland J.G. (2000). Comparison of left ventricular ejection fraction and volumes in heart failure by echocardiography, radionuclide ventriculography and cardiovascular magnetic resonance; are they interchangeable?. Eur. Heart J..

[29] The CONSENSUS Trial Study Group (1987). Effects of enalapril on mortality in severe congestive heart failure. Results of the Cooperative North Scandinavian Enalapril Survival Study (CONSENSUS). N. Engl. J. Med..

[30] Hjalmarson A., Goldstein S., Fagerberg B., Wedel H., Waagstein F., Kjekshus J. (2000). Effects of controlled-release metoprolol on total mortality, hospitalizations, and well-being in patients with heart failure: the metoprolol CR/XL randomized intervention trial in congestive heart failure (MERIT-HF). MERIT-HF Study Group. JAMA.

[31] Mcmurray J.J., Packer M., Desai A.S., Gong J., Lefkowitz M.P., Rizkala A.R. (2014). Angiotensin-neprilysin inhibition versus enalapril in heart failure. N. Engl. J. Med..

[32] Swedberg K., Komajda M., Bohm M., Borer J.S., Ford I., Dubost-Brama A. (2010). Ivabradine and outcomes in chronic heart failure (SHIFT): a randomised placebo-controlled study. Lancet.

[33] Gheorghiade M., Bohm M., Greene S.J., Fonarow G.C., Lewis E.F., Zannad F. (2013). Effect of aliskiren on postdischarge mortality and heart failure readmissions among patients hospitalized for heart failure: the ASTRONAUT randomized trial. JAMA.

